# Supplementary material for: Are carbon emissions trading and green financial instruments synergistic? -Comprehensive quantitative research based on content analysis
Source: PLoS One. 2024 Mar 7;19(3):e0298601. doi: 10.1371/journal.pone.0298601 (PMC10919601; doi:10.1371/journal.pone.0298601)
Supplement: S1 Data — (ZIP) [file pone.0298601.s001.zip › Data/地级市Z_F工作报告.zip!/╦─┤¿/├╝╔╜╩╨/└┤╫╘╣1⁄2╓┌║┼í╛┬φ┐╦╩2╛▌═°í┐_2017.docx]

尊敬的各位代表：

我代表市人民政府，向大会报告工作，请你们审查，并请列席会议的市政协委员提出意见。

2016年及过去五年工作回顾

2016年，市政府以供给侧结构性改革为主线，以项目投资为抓手，圆满完成了各项目标任务，实现了“十三五”良好开局。一年来的主要工作和成效是：

（一）着力稳定经济增长，发展质效迈上新台阶。2016年，全市地区生产总值（GDP）1117.23亿元，增长8.4%，居全省第3，上升1位，其中一二三产增加值分别增长4%、9.2%、9%，分别居全省第6、第5、第5。地方一般公共预算收入90.3亿元，同口径增长10%，居全省第8，上升5位。全社会固定资产投资1163.6亿元，增长12.7%，居全省第7，上升3位。社会消费品零售总额438.07亿元，增长12.7%，居全省第9，上升3位。城镇居民人均可支配收入28691元，增长8.7%，居全省第4，上升6位。农村居民人均可支配收入13935元，增长9.2%，总量全省第4。

（二）着力加快转型升级，产业能级实现新提升。规上工业增加值增长10%，居全省第5，“4+4”产业占工业比重达85%，战略性新兴产业占比提高2个百分点。电商交易额增长30%，成为第三届四川旅博会首个主题市，成功举办第13届中国旅游发展北京对话•七里坪峰会，新增国家4A级旅游景区3个，被亚太旅游协会评为新兴旅游目的地挑战项目（二三线城市）首选旅游目的地。“东坡味道”千亿产业实现销售收入670.26亿元，由农业部举办的全国果茶绿色发展经验交流会在丹棱召开，推广眉山发展柑橘经验新培育国家地理标志保护产品2个。

（三）着力推进改革创新，发展动能获得新增强。全面落实国务院“三去一降一补”政策，全年淘汰落后产能企业6户，新增上市挂牌企业13户，为企业减少成本9.39亿元。在全省率先举办工业企业闲置资产专题招商推介会，盘活资产70亿元。通过多方努力，帮助四川启明星铝业起死回生，实现8年以来的扭亏为盈。争取国省改革试点10项，农村居民住房公积金制度经验全国推广。申请发明专利555件，增长86.7%。新登记个体工商户、私营企业分别增长14.64%、23.75%。高端产业项目加快落地，引进华为、陶氏化学等世界500强、中国500强及投资10亿元以上项目26个，招商引资到位资金574.5亿元。成功举办第九届中国竹文化节、第八届中国泡菜博览会，吸引众多中外嘉宾，增强了眉山知名度。

（四）着力促进绿色发展，城乡面貌展现新形象。深入推进“三大工程”建设，森林覆盖率达48.61%，新增和恢复水域面积26平方公里，公园总数达118个。实施综合交通“千亿大会战”，完成投资120亿元。城镇建设完成222.9亿元，城镇化率达43.38%。中心城区空气质量优良率提升4.5个百分点。建成幸福美丽新村300个、省级“四好村”58个，93%以上村庄生活垃圾保持常态治理。洪雅建成国家生态县，青神县获国际竹藤组织“国际竹编之都” 称号和“竹编艺术传承国际范例奖”。

（五）着力抓好脱贫攻坚，民生福祉得到新改善。脱贫攻坚首战告捷，丹棱作为我省第一个县接受国家扶贫成效考核，受到高度评价。全年减少贫困人口4.6万人，4个省定、97个市定贫困村退出。建档立卡贫困人口在县域内定点医疗机构住院“零支付”，全省率先“两线合一”。十大民生工程、20件民生大事超额完成。仁寿县成功创建全国农民工返乡创业试点县，农村留守儿童排查工作经验全国推广。开工建设公办幼儿园12所，高考本科上线人数创历史新高。眉山籍运动员黄文攀里约残奥会打破5项世界纪录。市人民医院创建为三甲综合医院，全市达到血吸虫病传播阻断标准。东坡区残疾人和基层中医工作被评为全国先进。三苏祠博物馆全面修缮并重新开放，彭山江口沉银遗址水下考古取得阶段性成果。 “平安眉山” 建设深入推进，安全生产形势持续稳定向好。

（六）着力转变职能作风，政府建设取得新成效。深化简政放权，市本级取消行政审批67项，投资建设项目审批提速90%。整合开通党务政务服务热线，实现“一号一网”集中受理。完善政府常务会、专题会、每周例会等议事规则，建立市政府领导班子AB岗制度，政府办文时效平均缩短2天，政府工作更加务实、规范、高效。

2016年各项任务的完成，为市三届政府的工作画上了圆满的句号。回顾过去五年，全市上下抢抓机遇、奋力拼搏，团结一心、砥砺前行，谱写了眉山加快发展、跨越发展的新篇章。

（一）过去五年，是眉山产业加速集聚壮大、综合实力实现跃升的五年。新增50亿级企业1户，亿元以上企业超300户，国家高新技术企业达63户，建成百亿园区3个、百亿产业5个。现代产业体系正初步形成。GDP连跨五个百亿台阶，年均增长10.8%。地方一般公共预算收入是2011年的2.65倍。6个区县全部晋位升级。

（二）过去五年，是眉山改革开放不断深化、发展活力持续增强的五年。争取到国家、省级改革试点15项、30项，彭山农村土地流转等改革经验全国推广。民间投资占比64.7%，居全省第1。招商引资省外到位资金连续9年全省前3，是全省除成都以外落户世界500强企业最多的市州。

（三）过去五年，是眉山城市框架逐步拉开、城乡统筹深入推进的五年。城市规模扩大、品质提升，城镇化率从37.6%提高到43.38%。基础设施日趋完善，路网密度是全省的3.3倍。森林覆盖率比全国、全省分别高26.95、11.73个百分点。

（四）过去五年，是眉山社会事业迅速发展、公共服务全面提升的五年。拥有高校总数全省第3。医疗卫生服务体系建设全省领先。城市公共文明指数连续5年全省前3。连续5年无重大及以上事故发生。

（五）过去五年，是眉山民生保障日益完善、幸福指数不断提高的五年。全市全部财政性支出80%以上用于民生，701项民生承诺全部兑现。城乡居民收入分别比2011年增长66.9%、74.3%。贫困发生率从17.66%下降到0.78%。

各位代表，成绩来之不易，这是省委、省政府和市委坚强领导的结果，是市人大、市政协的依法监督、大力支持的结果，是全市人民团结奋斗的结果。在此，我代表市人民政府，向全市人民，向人大代表、政协委员、各民主党派、工商联、各人民团体、离退休老同志，向驻眉部队指战员、武警官兵、政法干警，向关心支持和参与眉山建设发展的社会各界人士，表示衷心的感谢并致以崇高的敬意！

在总结成绩的同时，我们也清醒看到当前发展中仍面临的困难和问题，主要是：经济总量不够大，产业结构不够优，创新能力不够强；城乡、区域之间发展不够均衡；优质教育、医疗供给不足，社会保障水平不够高，特别是生态环境保护与污染治理压力较大；部分政府人员 “能力不足、作风懈怠”仍不同程度存在。对上述问题，我们将高度重视，认真加以解决。

新一届政府努力方向

今后五年，新一届政府将紧紧围绕市第四次党代会确定的“决胜‘两个率先’，实现‘五个先行’，奋力建设天府新区增长极、大都市区新高地”目标，以清晰的思路、有力的措施，抓好工作落实，推动全市经济实力更强、城市品质更优、生态环境更美、人民生活更好。

（一）自觉践行创新发展理念，大力推进产业、科技、体制机制创新，推动经济发展更有活力、质量、效益、后劲，努力建成创新发展先行区。到2021年，GDP突破1800亿元，培育“五大千亿产业” “四个百亿产业”，科技进步对经济增长的贡献率超过60%，国家高新技术企业达100户。

（二）自觉践行协调发展理念，坚持世界眼光、国际标准、眉山特色，一体推进城乡规划、建设、管理，努力建成统筹城乡先行区。到2021年，全域融入成都和天府新区，建成宜居、宜业、宜商的生活品质之城，城镇化率达50%以上，80%以上新村建成省级“四好村”。

（三）自觉践行绿色发展理念，坚持生产、生活、生态三位一体，大力发展循环经济，加强污染治理，不断改善环境质量，加快建设天更蓝、地更绿、水更清的美丽眉山，努力建成生态文明先行区。到2021年，森林覆盖率达50%以上，新增和恢复水域面积100平方公里，新建公园42个，建成国家森林城市、联合国人居环境示范城市。

（四）自觉践行开放发展理念，围绕完善产业链、提升价值链，创新开展分层分类、定向定点招商，实施“六大千亿会战”，努力建成开放合作先行区。到2021年，招商引资到位资金累计达3600亿元，外商投资实际到位资金累计达6亿美元，全社会固定资产投资累计达8000亿元。

（五）自觉践行共享发展理念，始终以人民为中心，加快补齐教育、卫生等民生短板，不断增强人民群众安全感、获得感和幸福感，努力建成全面小康先行区。到2021年，城乡居民人均可支配收入分别达4万元、2万元以上，城乡居民基本养老、医疗保险覆盖率分别达95%、97%以上，人均预期寿命达77岁以上，群众社会治安满意度达85%以上。

2017年工作重点

今年是党的十九大和省第十一次党代会召开之年，是“两个率先”决胜年，是本届政府的开局之年，做好各项工作意义重大。

今年全市经济社会发展的主要预期目标是：GDP增长8%，全社会固定资产投资完成1300亿元，社会消费品零售总额增长12%，地方一般公共预算收入同口径增长8%，城乡居民人均可支配收入分别增长8%、9%，城镇登记失业率控制在4.3%以内，全面完成省定安全生产和节能减排目标。

为此，重点抓好五方面工作：

（一）聚焦创新引领，致力追求发展质量。

加快建设先进制造强市。深入实施“眉山制造2025”，推进智能制造、工业物联网、工业大数据创新应用等试点示范，实施“设备换芯”“生产换线”“机器换人”改造计划。加快推进中广核、陶氏化学等重点项目建设，完成工业投资460亿元、更新改造投资330亿元。力争规上工业增加值增长10%，战略性新兴产业产值增长15%，实现精细化工产值340亿元、机械及高端装备制造产值215亿元、新能源新材料及制品产值140亿元。

加快发展高效生态现代农业。推进农业农村“5+11”系统改革和农业供给侧结构性改革，实施农产品品牌战略，加快打造“东坡味道”千亿产业，实现销售收入740亿元。大力发展都市近郊型现代农业，“两城三园七片”完成现代农业投资65亿元。大力发展乡村旅游、农村电商等新产业、新业态、新模式，推动一二三产业融合发展。新培育省级示范合作社7家、家庭农场17家。推进竹编“双百工程”。粮食总产稳定在160万吨以上，打造全省粮食流通产业经济强市。一产增加值增长3.5%。

加快发展现代服务业。优先发展康养、电子商务、现代物流、现代金融、科技服务五大新兴先导型服务业，实施服务业“三百工程”，抓好97个重点项目建设，完成投资180亿元，三产增加值增长9%。推进全域旅游，加快黑龙滩、柳江古镇等重点景区建设，瓦屋山景区7月展示性开放，力争新增国家4A景区2个，实现旅游收入340亿元。推进35个重点文化产业项目建设，完成投资103亿元，建成省级文化产业园1个。

强化科技创新。实施企业创新主体培育工程，开展“三个一百”科技创新行动，新增省级科技企业孵化器2个、省级以上工程技术研究中心5个、省级企业技术中心1个，国家高新技术企业达65户，高新技术产业产值增长15%。深化校市合作。加强知识产权保护。完善人才政策，用好用活本土人才，引进一批急需紧缺高层次人才。

全面深化改革。推进“三去一降一补”，加快淘汰落后产能，妥善处置“僵尸企业”；支持居民自住需求和农民进城购房，促进房地产市场平稳健康发展；加强政府性债务管理，大力推广PPP模式，扩大企业直接融资，提高资产证券化率；支持 “小升规”“规改股”“股上市”，新增规上工业企业30户、上市挂牌企业20户；进一步降低企业税费负担和要素、物流成本，扩大直购电规模和直供气用户范围；启动全国质量强市示范城市创建，新增中国驰名商标1件、地理标志证明商标1件、国家地标产品1个、四川名牌25个。深化商事制度改革，力争新登记注册个体工商户1万户、私营企业2千户。扎实推进“3+N”重点领域改革落地落实。

（二）聚焦协调并进，致力打造新型城乡。

统筹城乡规划。强化顶层设计，推动“六规合一”。抓好新一轮眉山城市总规修编，加快土地利用总规调整，努力实现空间管控“一张蓝图”。抓好中心城区市政中心、太和、彭祖新城等片区控规编制和城市设计，完善天府新区眉山片区规划，完成眉山历史文化名城保护规划。

统筹城乡建设。投资200亿元实施“城镇基础设施建设推进年”行动，推进地下综合管廊、城区路网等304个项目建设，完成东坡大道改扩建等工程。完成国家新型城镇化综合试点任务，加快100个特色乡镇建设，优先建设13个国省重点镇，常住人口、户籍人口城镇化率分别达45%、32.5%。实施“千亿交通大会战”，构筑贯穿全城、辐射全域的“三横三纵”千里大通道，力争新开工高速公路2条、一级公路3条、航电工程3个，加快天府大道眉山段建设，完成剑南大道眉山段建设，成昆铁路眉山段完成扩能改造，蒲简、仁沐高速眉山段竣工通车，新（改）建农村公路500公里，完成投资130亿元。统筹城乡水利、能源、信息基础设施建设，推进全域安全饮水、全域污水治理、全域垃圾处理。实施“厕所革命”行动，新（改）建A级旅游厕所40座、公共厕所100座以上，让游客、市民更“方便”。

统筹城乡管理。在抓好城建“大项目”的同时，始终不忘群众“身边事”，以旅游城市标准推进城乡精细化管理，让城乡居民享受更便利、更暖心的服务。加强市政设施管养维护，改造锈蚀路灯188组。大力发展绿色交通，新投放新能源公交车40辆、公共自行车1500辆，新建公交候车亭56个。开展城市堵点综合整治，缓解城区拥堵和“停车难”。开展违法建设专项整治，严控新增“两违”。启动国家卫生城市创建。坚持乡村环境治理与乡村旅游相结合，推进120个幸福美丽新村建设，创建省级“四好村”100个。

（三）聚焦美丽眉山，致力优化生态环境。

加强生态建设。启动四川省和国家森林城市创建，加强生态绿地系统建设和保护，完成营造林6万亩，城乡绿化覆盖率达52%。加快城区“四湖”建设，启动大东坡湿地、黑龙滩优良湖泊建设，恢复和新增水域面积20平方公里。完成东坡城市湿地公园二期建设，新建成公园10个。

狠抓环境整治。打好污染防治三大战役，实施“点长”治气，抓好工业废气、扬尘、机动车、生物质无序焚烧和餐饮油烟污染治理，中心城区PM2.5年均浓度下降9.9%、空气优良天数率66.3%；坚持“工业污水不出园（厂）、种养污水不入河、生活污水全进管”，实施“河长”治水，推进球溪河等17条河流治理，开工建设通惠河醴泉河污染综合治理项目，全面消除中心城区黑臭水体；实施“田长”治土，加强农村面源污染防治，完成非正规垃圾堆放点排查整治，畜禽粪污综合利用率达70%。加大环保督察和监管力度，抓好环保突出问题整改。加大环境执法与刑事司法联动力度，从快从严从重打击环境犯罪行为。建立以亩均效益为核心的资源集约利用正向激励和反向倒逼机制，开展能源和水资源消耗、建设用地等总量和强度双控行动。

（四）聚焦招强选优，致力扩大开放合作。

精准促进投资。围绕“五大千亿、四个百亿”产业集群，紧盯世界、中国、民企500强以及跨国公司和行业龙头企业，开展精准招商、选商，着力引进一批标杆企业和优质项目，加快形成新的经济增长点和产业支撑。加快推进项目落地建设，完善征地拆迁机制，强化要素保障，尽快将意向性项目转化为实际投资。招商引资到位资金620亿元，引进世界、中国、民企500强或投资10亿元以上项目20个。

提升开放平台。“一园一策”推动产业园区转型升级、创新发展，培育壮大6个百亿园区，启动建设眉山空港经济区，推进眉山经开区创国家经开区、中国泡菜城创国家农业高新区、视高园区创省级高新区，争创省级产业园5个。高水平办好第八届东坡文化节、第九届中国泡菜博览会、中外知名企业眉山行、“东坡•味道”全球行。

深化区域合作。深化成眉合作，召开成眉同城发展高层会议，打通与成都无缝对接的快速通道，使眉山更快更好融入成都。深化甘眉、雅眉、乐眉合作。加强与长三角、泛珠三角、京津冀和港澳台等地区合作。

（五）聚焦脱贫攻坚，致力增进民生共享。

决战决胜脱贫攻坚。围绕“两不愁、三保障”和“四个好”目标，突出产业扶贫，精准推进一村一品、一乡一业，易地扶贫搬迁9962人，19548名贫困人口脱贫，30个市级贫困村退出，东坡区、仁寿县、洪雅县完成脱贫任务。全面巩固脱贫成果。抓好阿坝州金川县、茂县对口帮扶。

切实保障改善民生。坚持民生投入和为老百姓办实事只增不减，全部新增财力和八成以上财政性支出用于民生。从解决群众普遍关心的突出问题入手，继续实施十大民生工程和20件民生大事，完成投入150亿元。抓好高校毕业生等重点人群就业创业，新增城镇就业2.6万人。实施全民参保计划，落实阶段性降低社会保险费率政策。改造城镇危旧房棚户区10050户、农村危房637户。

全面发展社会事业。统筹各级各类教育协调发展，提升教学质量和教育水平。实施全市学校、体育场馆布局规划，新（改、扩）建校舍5万平方米。实施中心城区教育扩容工程，加快苏辙中学、东坡岛小学建设，启动建设西北片区和齐通路幼儿园、小学、中学等6所学校，力争5年内中心城区17个教育体育项目全面建成，基本解决“就学难”问题。启动市区体育中心建设，完成“体育两中心”主体工程，办好2017中国国际友好城市青少年足球邀请赛。全面推进“健康眉山”建设，加强城乡医疗卫生计生基础设施和服务能力建设，加快市中医院迁建，市人民医院二期完成主体工程，力争开工市妇幼保健院等中心城区10个医疗卫生机构建设项目，新（改、扩）建一批县级医疗卫生机构，新建乡镇卫生院5个，力争5年内基本解决“看病难”问题。加强师德师风、医德医风建设。完善公共文化服务体系，建设城市“十五分钟文化圈”和农村“十里文化圈”，加强文物普查、保护，启动眉山古城墙和太和镇历史文化街区建设整治。推进各类养老服务体系建设。完成市儿童福利院建设。统筹抓好统计、外侨台、民族宗教、地方志、保密、档案、气象、测绘、供销、红十字、慈善、妇女、儿童、关心下一代等工作。

建设平安和谐眉山。全面落实“党政同责、一岗双责、齐抓共管、失职追责”的安全生产责任体系，坚决遏制重特大事故发生。加大“双安双创”力度，切实保障群众“舌尖上的安全”。加强应急管理，抓好洪涝、地质灾害等防灾减灾工作。加快构建立体化、信息化社会治安防控体系，常态化开展打击“两抢一盗”专项行动，黑恶势力打击率、现行命案侦破率100%。深入推进依法治市，抓好“七五”普法，深化网格化管理，支持工会、共青团、妇联等群团和社会组织参与社会治理。健全信用体系，加强政务诚信、商务诚信、社会诚信和司法公信建设。深化“走基层”活动，扎实开展“五必送、五必访”。加强国防动员和民兵预备役工作，做好人民防空、交通战备和征兵工作。

建设“实干、高效、廉洁”的人民满意政府

新目标、新任务，对政府自身建设提出了新的更高要求。我们要牢固树立“四个意识”，坚决落实全面从严治党要求，扎实推进从严治政、依法行政、廉洁从政，努力使政府工作更有效、政府形象更良好、人民群众更满意。

各位代表！

蓝图已绘就，关键在落实。我们将始终保持实干、苦干、拼命干的劲头，弘扬求真务实精神，着力增强狠抓落实的自觉性和责任感，勇挑大梁、不计名利、夙夜在公，把全部心思用在干实事、求实效上。严格落实岗位责任制、督查督办制、考核问责制，增强行政执行力，确保中央、省和市各项决策部署不折不扣落到实处。量化、细化、项目化政府工作，以钉钉子精神抓落实、抓攻坚，确保各项工作落细、落小、落地。健全正向激励和容错纠错机制，让广大干部想干事、敢干事、干成事。

我们将全力推进速办、快办、网上办，不断提高政府工作效率。围绕降低制度性成本，深化“放管服”改革，优化流程、精简环节、减少程序，清理隐形审批和变相审批，强化网上审批、并联审批和集中审批，推进“多证合一”“多评合一”，推行容缺受理、提前审核和延时服务、预约服务、上门服务、微笑服务制度，实现效率再提升、服务再优化。全面推行“互联网+政务服务”，逐步实现“一号申请、一窗受理、一网通办” ，让企业和群众办事更省时、省心、省钱。全面推行AB岗制度，严格首问负责制。强化眉山政务云平台和眉山市经济社会综合信息平台的管理应用，推进政府系统无纸化办公。

我们将努力做到心廉、行廉、保真廉，确保政府始终廉洁奉公。推进“两学一做”学习教育常态化制度化，加强廉政建设和作风建设，严格落实“两个责任”，持续整治“庸懒散浮拖”。深入实施《法治政府建设实施纲要》，推进依法科学民主决策，严格规范公正文明执法，加强和改进行政复议、行政应诉工作，做尊法守法的表率。习惯在监督下开展工作，主动接受市人大及其常委会的法律监督、工作监督和市政协的民主监督，认真执行市人大及其常委会决议决定，每年向市人大或其常委会专项报告环境状况和环境保护目标完成情况，高质量办好人大代表建议和政协提案；高度重视司法、舆论和社会监督，加强审计监督，及时整改问题，不断健全制度、堵塞漏洞；深化政务公开，确保权力在阳光下廉洁运行。

各位代表！光荣的使命，鼓舞我们砥砺奋进；人民的期盼，鞭策我们勇往直前。让我们紧密团结在以习近平同志为核心的党中央周围，在市委的坚强领导下，与全市人民一道，开拓进取，全力拼搏，决战决胜“两个率先”，为建设天府新区增长极、大都市区新高地而努力奋斗！
